# Extracellular vesicle GABA responds to cadmium stress, and GAD overexpression alleviates cadmium damage in duckweed

**DOI:** 10.3389/fpls.2025.1536786

**Published:** 2025-03-18

**Authors:** Zhanpeng Sun, Ziyang Qu, Yuman He, Yujie Han, Yun Xing, Sizheng Liu, Yi Hu, Yumeng Jiang, Yiqi Yu, Yuanyuan Liu, Weibo Sun, Lin Yang

**Affiliations:** ^1^ Tianjin Key Laboratory of Animal and Plant Resistance, College of Life Sciences, Tianjin Normal University, Tianjin, China; ^2^ Faculty of Education, Tianjin Normal University, Tianjin, China; ^3^ School of Life Sciences, Westlake University, Hangzhou, China; ^4^ Tsinghua-Peking Center for Life Sciences, College of Life Sciences, Tsinghua University, Beijing, China

**Keywords:** duckweed, cadmium, GABA, GAD, extracellular vesicles, abiotic stress

## Abstract

**Introduction:**

Cadmium (Cd) pollution lead to ecological problems and cause severe damages to plants. Investigating the signal response to Cd is crucial for improving Cd resistance during phytoremediation. While γ-aminobutyric acid (GABA) is known to accumulate rapidly under environmental stress, the real-time dynamics of GABA signaling and its mechanistic link to stress adaptation remain poorly understood.

**Methods:**

In this study, a sensitive GABA biosensor, iGABASnFR, was introduced into plants for the first time to monitor GABA signaling. Additionally, glutamate decarboxylase (GAD), a key enzyme catalyzing the conversion of glutamate (Glu) to GABA, was overexpressed in duckweed. The responses of GABA in extracellular vesicles (EVs) under Cd stress were analyzed using iGABASnFR transgenic duckweed. Cd accumulation, photosynthesis, and antioxidant activity were evaluated in GAD-overexpressing duckweed.

**Results:**

(1) GABA in extracellular vesicles of duckweed exhibited a dynamic response to Cd stress, as visualized by iGABASnFR transgenic duckweed. GABA content in EVs was significantly enhanced under Cd treatment. (2) GAD-overexpressing duckweed demonstrated improved photosynthetic efficiency and enhanced antioxidant capacity during Cd stress. (3) Cd accumulation was significantly increased in GAD transgenic duckweed, as evidenced by Cd^2+^ flux measurements, total Cd content, and Cd staining in protoplasts using FlowSight imaging.

**Discussion:**

This study provides novel insights into the role of GABA in extracellular vesicles during Cd stress and establishes a direct link between GABA signal and Cd stress adaptation. The findings demonstrate that GAD overexpression enhances Cd resistance and accumulation in duckweed, offering a potential strategy for improving phytoremediation efficiency. This work advances our understanding of GABA signaling dynamics and its application in Cd stress.

## 1 Introduction

Cadmium (Cd), a toxic heavy metal, poses serious threats to both the ecological environment and human health (Wang et al., 2021). Cd enters into aquatic systems, such as rivers and lakes, through various anthropogenic activities such as mining, industrial emissions, and battery manufacturing, as well as through chemical fertilizers (Afzaal et al., 2022). Cd decreases chlorophyll content and prevents photosynthesis in aquatic plants and algae, impacting primary productivity and disrupting food stains. In addition, excessive Cd accumulation reduced nutrient uptake, inhibited plant growth and respiration, and changed antioxidant systems and cell membrane function (Shahid et al., 2017). Furthermore, Cd bioaccumulates in aquatic organisms, leading to reproductive failures, growth retardation, and even death, thereby threatening biodiversity and the overall health of aquatic ecosystems (Yang Q. et al., 2023). When Cd enters the human body through ingestion of contaminated food or water, it can cause a wide range of adverse health effects (Bakulski et al., 2020). It can accumulate in the brain, trigger neurotoxic responses, and increase the risk of neurodegenerative diseases like Alzheimer’s disease (Deng et al., 2024). Therefore, addressing Cd contamination in aquatic environments has become a global priority. While wastewater remediation technologies can partially reduce Cd levels, preventing contamination at its source through stricter regulations and sustainable industrial practices remains the most cost-effective and ecologically viable strategy. Addressing Cd contamination in aquatic environments has become a global priority. Phytoremediation has emerged as a promising and eco-friendly approach (Ma and Jiang et al., 2024) to remediate Cd polluted water water conditions.

Duckweed (*Lemnaceae*) is the smallest flowering vascular plant and one of the fastest-growing plants, with a doubling time of approximately 48 h (Romano et al., 2024). There are several advantages of employing duckweed in Cd remediation: 1) Duckweed is the key component of natural ecosystems and plays a significant role in maintaining aquatic ecological balance (Acosta et al., 2021). Duckweed provides food for aquatic animals and participates in the material cycle of natural ecosystems. In addition, duckweed can absorb a large amount of Cd in water, thereby reducing the content of Cd in water and its harmful effects on aquatic animals. Duckweed can fix carbon and nitrogen through photosynthesis, but Cd pollution will affect the growth of duckweed, affect the carbon and nitrogen cycle, and destroy the aquatic ecological balance. 2) Duckweed shows high growth rates and elevated photosynthetic efficiency ability (Stewart et al., 2021). 3) Duckweed exhibits immense potential according to studies conducted on bioenergy (Chen et al., 2022), rhythm (Muranaka and Oyama, 2016), and signal (Yang et al., 2022), establishing its role in environmental remediation. Hence, a number of studies have focused on Cd accumulation by duckweed. However, phytoremediation has inherent limitations. Plants can accumulate substantial amounts of Cd in their tissues, which might be consumed indirectly by animals, and through these, heavy metals may be introduced into the environment, resulting in secondary pollution and significant health risks (Ali et al., 2013). Moreover, Cd is highly toxic to most living organisms (Yan et al., 2023). In plants, Cd stress severely affects growth and productivity, often leading to reduced biomass and disruptions in the photosynthetic system (Marques et al., 2023). These challenges limit the effectiveness of Cd adsorption in phytoremediation. Therefore, it is imperative to elucidate the Cd signaling mechanisms in response to cadmium toxicity, in order to develop Cd-tolerant and high Cd-accumulating duckweed strains for effective and sustainable phytoremediation applications.

Plants, unlike animals which can move, sense local stimuli by highly sensitive signaling systems to adapt and survive in their environments. In animals, the amino acid glutamate (Glu) is an excitatory neurotransmitter, which also plays as a wound signal in plants (Toyota et al., 2018). Gamma-aminobutyric acid (GABA), an inhibitory neurotransmitter, is the interconvertible metabolism with Glu, which plays a pivotal role in maintaining neurotransmitter homeostasis and regulates various plant physiology processes such as the modulation of stomata (Xu et al., 2021). The conversion of L-glu to GABA primarily occurs via the action of the enzyme glutamate decarboxylase (GAD), which catalyzes the decarboxylation of L-glu, removing a carboxyl group to produce GABA and carbon dioxide (Michaeli and Fromm, 2015). In our previous studies, the Glu content was increased under Cd stress, which triggered a Ca^2+^ signal in duckweed. Furthermore, GABA level decreased with 24-h Cd treatment, indicating a disruption in its normal synthesis or degradation pathways. The addition of GABA increased the abscission rate, while the addition of Glu decreased it during Cd stress, further underscoring the intricate interplay between these molecules and their roles in the plant’s stress response (Yang et al., 2020). Therefore, the signaling function of Glu is not only relevant to Cd stress but also intricately linked to GABA metabolism, collectively influencing the plant’s ability to cope with Cd stress.

GABA leads to the rapid efflux of malate anions through the aluminum-activated malate transporter (ALMT) family (Ramesh et al., 2015). Scientists showed that GABA signals modulate plant growth and response to environmental stress (Jin et al., 2023). The mechanisms of GABA perception and signaling in plants include 1) GABA-responsive enzymes: GABA modulated the activity of enzymes involved in stress responses or metabolic pathways, which suggested that GABA played a role as a “stress memory” in plants during stress (Ji et al., 2020). 1) GABA receptors: Xu found that under a water deficit, GABA concentration increased and reduced stomatal opening in an ALMT9 (a GABA receptor)-dependent manner (Xu et al., 2021). 3) Second messenger systems: GABA application in plants modulates second messenger systems, such as calcium signaling or reactive oxygen species (ROS) production (Khalil et al., 2024). IV) Transporters and channels: GABA transporters and channels are involved in the uptake, extrusion, or intracellular distribution of GABA in plants (Meier et al., 2024). 5) Crosstalk with other signaling pathways: GABA signaling in plants may intersect with other hormonal or stress-responsive pathways, such as those mediated by abscisic acid (ABA), ethylene, or jasmonic acid (Kabała and Janicka, 2024). This crosstalk enables plants to incorporate GABA signals into broader regulatory networks influencing growth, development, and stress responses. However, the GABA perception and signaling in Cd stress remains unknown.

Here, the specific aims of our study are to 1) investigate how GABA responds during Cd stress by the sensitive GABA sensor iGABASnFR transgenic duckweeds; 2) examine the role of GABA in extracellular vesicles during Cd stress; 3) assess Cd resistance and accumulation in GAD transgenic duckweed; and 4) gain a comprehensive understanding of GAD modulating Cd stress tolerance, particularly at the level of ion homeostasis, amino acid metabolism, and antioxidant responses.

## 2 Materials and methods

### 2.1 Plant material culture

Duckweed (*Lemna turionifera* 5511) was firstly taken from the Fengchan River of Tianjin and subsequently cultivated in our laboratory for 16 years. The culture medium contains 0.4 mM of MgSO_4_·7H_2_O, 1.4 mM of Ca(NO_3_)_2_·4H_2_O, 1.0 mM of KNO_3_, 0.4 mM of KH_2_PO_4_, 0.4 mM of Mg(NO_3_)_2_·6H_2_O, 50 μM of CaCl_2_·2H_2_O, 50 μM of KCl, 6.1 μM of Na_2_MoO_4_·2H_2_O, 69 μM of H_3_BO_3_, 30 μM of K_2_H_2_EDTA·2H_2_O, 56.7 μM of FeNH_4_-EDTA, 13.8 μM of MnCl_2_·4H_2_O, 2.8 μM of ZnNa_2_EDTA·4H_2_O, 4.8 μM of CoSO_4_·7H_2_O, and 18.6 μM of Na_2_-EDTA·2H_2_O (Ma et al., 2024). The medium pH was adjusted to 5.8, followed by steam sterilization at 121°C for 20 min. The duckweed was cultured under 16 h light/8 h dark cycles, with an intensity of 95 μmol m^−2^ S^−1^, and the temperature was kept at 24°C day/22°C night.

Duckweed was treated with or without 50 μM of CdCl_2_ for 24 h, and the root abscission rate was calculated using the following formula: Number of roots abscised/Total number of roots × 100%.

### 2.2 Plasmid construction and generation of transgenic duckweed

**Figure 1:**
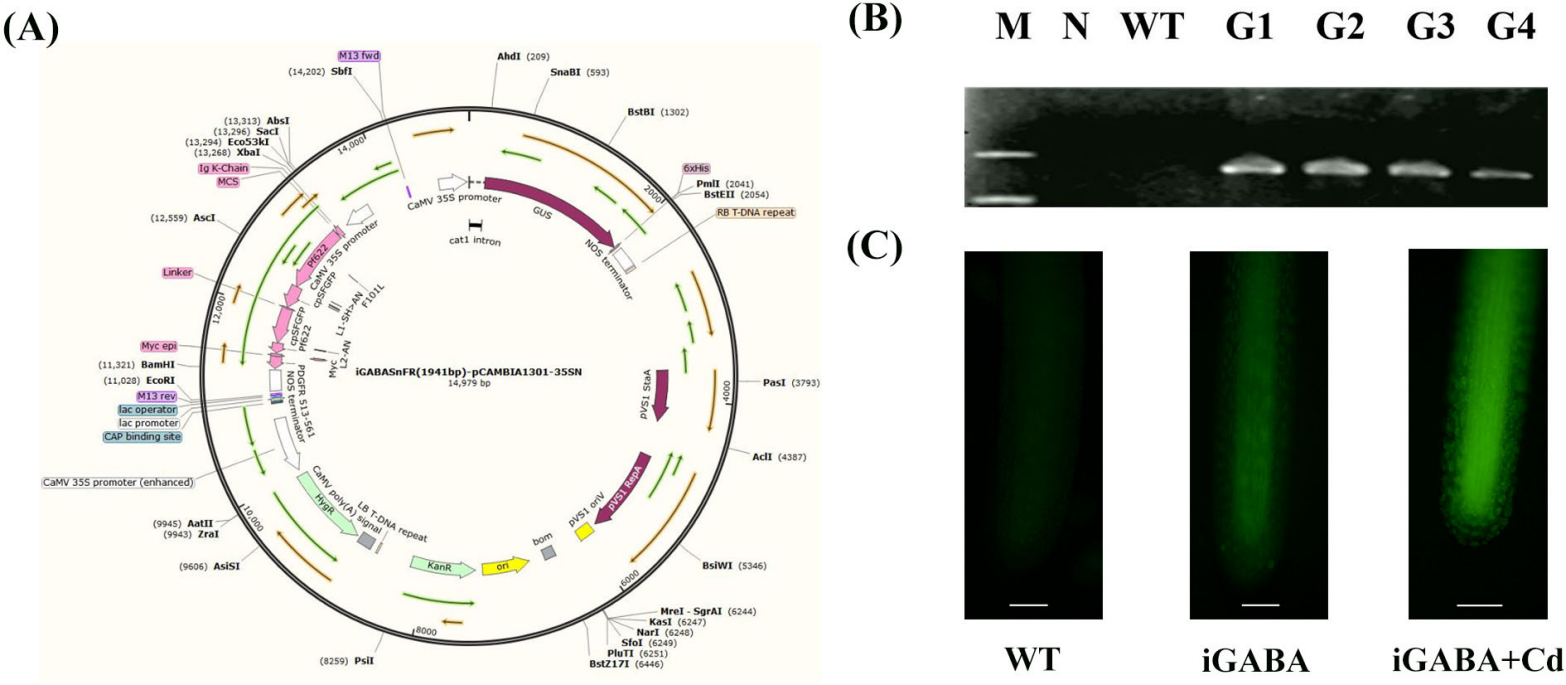
**(A)** Construction of pCAMBIA-1301-iGABASnFR. KanR, kanamycin resistance; HygR, hygromycin resistance; GUS, β-glucuronidase. **(B)** Specific PCR amplification for *iGABASnFR* identification. N, negative control (where water served as the template for PCR); WT, wild type; G1-4, *iGABA*1-4. **(C)** Fluorescence signal diagram of iGABA under Cd stress.

The GFP-based GABA-sensing fluorescent reporter iGABASnFR, firstly constructed by Marvin et al. (2019), was connected by the cauliflower mosaic virus 35S (CaMV-35S) promoter at the 5′ end and the terminator of the nopaline synthase gene (NOS) at the 3′ end. Then, it was inserted into the binary vector pCAMBIA 1301 (**Figure 1A**). The *AtGAD* gene was obtained from *Arabidopsis thaliana* through reverse transcription. The pCAMBIA 1301-*AtGAD* was constructed by inserting the target gene *AtGAD* between the CaMV 35S promoter and the NOS terminator using PCR and restriction enzyme digestion with *Nco*I and *BstE*II at 37°C. Both of the vectors were transformed into the *Agrobacterium tumefaciens* strain EHA105.

Duckweed callus was induced from explants for *Agrobacterium-*mediated transformation: 10 to 15 days of fully expanded duckweed was selected, the root was removed, and the frond was scratched horizontally. Subsequently, the explants were transferred to the B5 medium with 1.5% sucrose, 15 mg/L of dicamba, 3.5 mg/L of 2,4-dichlorophenoxyacetic acid (2,4-D), and 1 mg/L of 6-benzyladenosine (6-BA). After 2–3 weeks of induction, the callus was transferred to a B5 culture medium with 1.5% sucrose, 10 mg/L of 4-chlorophenoxyacetic acid (CPA), and 2 mg/L of 6-(γ,γ-dimethylacrylamide)-purine (2ip) for subculture (Ma et al., 2024). Callus transformation was then performed following the method described by the *Agrobacterium*-mediated method (Ma et al., 2024; Yang et al., 2017). Hygromycin was used for the selection of transformed plants.

### 2.3 PCR analysis

The DNA of *iGABASnFR* and *GAD* transgenic duckweed was extracted using the Plant Genomic DNA Kit (TIANGEN, Beijing, China). A 20-µL PCR mixture was prepared, containing 10 µL of Premix Taq (TaKaRa Taq™ Version 2.0), 2 µL of template, 1 µL of forward primer, 1 µL of reverse primer, and 6 µL of ddH_2_O, followed by 30 cycles at 94°C for 1 min, 58°C for 45 s, and 72°C for 1 min 30 s. The primers used were as follows:

F 5′-CCATGGTAATGGTGCTCTCCCACGCC-3′

R 5′-GTACCTTAGCAGATACCACTCGTCTT-3′ (*GAD*)

F 5′-TGAACGATCTGCAGCCCGGGGGATCC-3′

R 5′-TCCACCGCGGTGGCGGCCGCTCTAGA-3′ (*iGABASnFR*)

### 2.4 Fluorescence microscopic observation of iGABA duckweed

The *iGABASnFR* duckweed (iGABA) was collected in a 1.5-mL EP tube, soaked in 20 mM of Na_2_-EDTA for 10 min, rinsed with ddH_2_O two to three times, added with 100 µL of FM4-64 Dye (Molecular Probes, Invitrogen, Carlsbad, California, United States) at a final concentration of 10 µM, and washed three times with 0.01 M of phosphate-buffered saline (PBS) (pH = 7.4). An upright fluorescence microscope was used to observe the treated duckweed (Leica DFC450C, DM5000, Berlin, Germany).

### 2.5 Extraction and observation of PDEVs

Twenty grams of duckweed (fresh weight) was treated with a high-speed homogenate in 300 mL of PBS to obtain the supernatant. The supernatant was collected by centrifugation at 4°C, 1,000×*g* for 10 min, 2,000×*g* for 20 min, 4,000×*g* for 30 min, and 10,000×*g* for 60 min. Then, the supernatant was absorbed carefully and the large particle vesicles (larger than 220 nm) were removed with a sterile 0.22-µm filter. Subsequently, the filtered supernatant was centrifuged at 120,000×*g* for 70 min at 4°C, and then the supernatant was removed. The plant-derived extracellular vesicle (PDEV) precipitate was resuspended with 50 µL of PBS and transferred to a 1.5-mL EP tube for the subsequent experiment or stored at −80°C. PDEV samples (20 µL) were sucked into a copper net for 5–10 min. A pipette absorbed 20 µL of 2% phosphotungstic acid solution. Extra droplets were removed by suction using filter paper and dried under an incandescent lamp. The PDEV samples were observed and photographed under a transmission electron microscope (HITACHI, HT7700, Japan) following Yang L. et al. (2023). PDEVs were diluted in sterile PBS, filtered through a 0.22-μm filter, and tested by nanoparticle tracking analysis (NTA) (Malvern Panalytical NanoSight, NS300, Great Malvern, Worcestershire, England).

### 2.6 Western blot analysis of TET8 in PDEVs

Proteins (20 μg per lane) were separated on 12% SDS-PAGE gels and transferred to PVDF membranes. Membranes were blocked with 5% non-fat milk in TBST (Tris-buffered saline with 0.1% Tween-20) for 1 h. The primary antibodies—rabbit anti-tetraspanin 8 (TET8) (1:1,000, Agrisera) and mouse anti-H+-ATPase (1:5,000, plasma membrane contamination control)—were incubated overnight at 4°C. The secondary antibodies—HRP-conjugated goat anti-rabbit IgG (1:5,000) and anti-mouse IgG (1:5,000)—were incubated for 1 h at room temperature. Signals were detected using an ECL substrate and visualized with a ChemiDoc system.

### 2.7 Determination of the GABA content of duckweed PDEVs

To determine the GABA content in duckweed PDEVs under Cd stress, ultra-performance liquid chromatography-mass spectrometry/mass spectrometry (UHPLC-MS) analyses were performed using a Vanquish UHPLC system (Thermo Fisher, Waltham, MA, USA) coupled with an Orbitrap Q Exactive™ HF mass spectrometer (Thermo Fisher, Waltham, MA, USA). Samples were injected into a Hypersil Gold column (100 × 2.1 mm, 1.9 µm) using a 17-min linear gradient at a flow rate of 0.2 mL/min. The eluents for the positive polarity mode were eluent A (0.1% formic acid in water) and eluent B (methanol). The eluents for the negative polarity mode were eluent A (5 mM ammonium acetate, pH 9.0) and eluent B (methanol). The solvent gradient was set as follows: 2% B, 1.5 min; 2%–85% B, 3 min; 85%–100% B, 10 min; 2%–100% B, 10.1 min; and 2% B, 12 min. A Q Exactive™ HF mass spectrometer was operated in positive/negative polarity mode with a spray voltage of 3.5 kV, a capillary temperature of 320°C, a sheath gas flow rate of 35 psi, an aux gas flow rate of 10 L/min, an S-lens RF level of 60, and an aux gas heater temperature of 350°C.

### 2.8 Determination of chlorophyll content

The chlorophyll content of duckweed was determined after being treated with 50 μM of CdCl_2_ for 48 h. A total of 0.2 g of duckweed was collected and immersed in 25 mL of 95% alcohol for 24 h. The absorbance of the extract was measured at wavelengths of 645 nm and 663 nm using a multimode microplate reader (TECAN, Spark^®^ Multimode Microplate Reader, Mannedorf, Switzerland) to calculate the chlorophyll content. Chlorophyll a (Chl a), chlorophyll b (Chl b), and total chlorophyll (Chl a+b) were calculated as follows: (Sumanta et al., 2014)


Chl a (mg/g)={(12.7A663-2.69A645) × 25 ml}/0.2 g

Chl b (mg/g)={(22.9A645-4.68A663) × 25 ml}/0.2 g

Chl(a+b)(mg/g)=Chl a+Chl b

### 2.9 RNA sequencing and analysis

The RNA from wild-type (WT) and *GAD* transgenic duckweed (GAD) was extracted and analyzed at Novogene (Chaoyang, Beijing, China). Total RNA was collected using the RNA prep Pure Plant Kit (TIANGEN, Beijing, China). The quality of the RNA was assessed using an Agilent 2100 Bioanalyzer (Santa Clara, CA, USA). Subsequently, the library was constructed and examined using the Illumina NovaSeq 6000 sequencing system (San Diego, CA, USA). The samples were subjected to gene expression analysis using transcriptomic methods at Novogene (Chaoyang, Beijing, China). The raw sequencing data obtained were processed using standard bioinformatics pipelines, involving quality control checks, trimming of low-quality sequences, and mapping of the clean reads to the reference genome. For the differential gene expression analysis, the DESeq2 software was employed to compare the read counts of each gene across the samples and determine which genes were significantly up- or downregulated. Additionally, the functional annotation tools such as Gene Ontology (GO), Kyoto Encyclopedia of Genes and Genomes (KEGG), and pathway analysis were used to gain the difference of the biological processes and pathways that are affected by Cd stress and determine the role of GABA and GAD in modulating these responses. Gene functional annotation was performed using the following databases: GO, the KEGG Ortholog (KO), and the manually annotated and reviewed protein sequence database (Swiss-Prot). Expression levels were analyzed following Li and Dewey (2011).

### 2.10 Protective enzyme activity assay

Superoxide dismutase (SOD), peroxidase (POD), and catalase (CAT) were determined by the double-antibody sandwich enzyme-linked immunosorbent assay (ELISA) kit (Enzyme-linked Biotechnology Co., Shanghai, China). A 0.1-g duckweed (fresh weight) was ground in liquid nitrogen. Subsequently, 1 mL of PBS was added to it and collected using an EP tube. The supernatant was collected after centrifugation at 4°C, 12,000×*g* for 2 min. As a sample, the supernatant was diluted five times. The purified plant SOD/POD/CAT antibodies were coated on microporous plates to create the solid-phase first antibody. The diluted supernatant was added to the microporous plate and incubated at 37°C for 30 min. After washing it five times with a washing solution, the horseradish peroxidase (HRP)-labeled detection antibodies were added to form an antibody–antigen–enzyme-conjugated antibody complex, incubated for 30 min, and washed again with the washing solution. Finally, 3,3′,5,5′-tetramethylbenzidine (TMB) was added for coloration and the absorbance (OD) was measured at a wavelength of 450 nm using an enzyme-labeled instrument (TECAN, Spark^®^ Multimode Microplate Reader, Seestrasse, Mannedorf, Switzerland).

### 2.11 Determination of Cd content in the medium

WT and GAD were cultured with 50 mL of CdCl_2_ solution (50 µM) for 24 h. After the liquid medium was acidified with 3% nitric acid, the Cd content of the solution was measured using an inductively coupled plasma emission spectrometer (ICP, Agilent ICP-OES 725 ES, CA, USA).

### 2.12 Cd^2+^ flux determination

Both WT and GAD were exposed to 50 µM of CdCl_2_ for 30 min. The non-invasive microtest technique (NMT Physiolyzer^®^, Younger, Amherst, MA, USA) was employed to measure the net Cd^2+^ flux in the root tip cells and mesophyll cells of WT and GAD by the Younger USA NMT Service Centre (Xuyue, Beijing, China). The NMT test solution consisted of 0.1 mM of CaCl_2_, 0.1 mM of KCl, 0.3 mM of MES, and 50 µM of CdCl_2_ at pH 5.8. The Cd^2+^ flux in the roots (100 μm to the root apex) and mesophyll cells was measured for 702 s by a Cd^2+^ selective microsensor between two points near (2 μm) the surfaces of the roots and fronds repeatedly.

### 2.13 Extraction of the protoplasts and flow cytometric analysis

Duckweed was soaked in 95% ethanol for 15 min, and the roots and fronds were separated. Subsequently, they were incubated in the dark at 37°C for 60 min with a mixture of 1% cellulase and 1% pectinase. The supernatant was collected after centrifugation at 4°C, 6,000×*g* for 5 min to get the protoplasts that were rinsed three times using Dulbecco’s phosphate-buffered saline (DPBS). After that, the protoplasts were stained in the dark with 30 μL of Leadmium™ Green AM dye (100 μg/mL) at 37°C for 60 min and washed three times with DPBS. After being filtered using a 200-mesh cell filter, the intracellular Cd content was analyzed using FlowSight (Merck Millipore, FlowSight^®^ Imaging Flow Cytometry, Darmstadt, HE, Germany).

### 2.14 Statistical analysis

All experiments were repeated with six replicates, with more than 20 fronds per group of parallel experiments for fluorescence microscopic observation, 0.5 g of duckweed with three replicates for gene expression study and five replicates for Cd^2+^ flux determination, and 6,000 protoplasts with three replicates for flow cytometric analysis. Experimental data were organized using Microsoft Excel 2019 and plotted using GraphPad Prism 9.3 and Adobe Illustrator 2023. Variables were subjected to the independent sample tests and one-way ANOVA in SPSS software (IBM SPSS Statistics, Version 26). Significant differences are indicated by asterisks (*p < 0.05, **p < 0.01, ***p < 0.001).

## 3 Results

### 3.1 Construction and identification of iGABA transgenic duckweed

The recombinant vector was constructed as described in **Figure 1A**. Subsequently, genomic DNA was extracted from the plants and subjected to specific PCR amplification targeting the *iGABASnFR* gene for identification purposes. Significantly, iGABA was not amplified in the negative control or WT. These results confirmed the successful transformation of all four transgenic strains (**Figure 1B**). The expression of GAD was tested by RNA sequencing, and the results in **Supplementary Table S1** showed that the expression of GAD was 5.67 log_2_ fold change than that of WT duckweed, which is statistically significant (*p* = 1.5 * 10^−8^). iGABA exhibited a significantly stronger fluorescence signal in response to CdCl_2_ treatment. This observation directly supports the claim that iGABASnFR transgenic duckweed exhibits a notable increase in GABA fluorescence signals in response to cadmium stress (**Figure 1C**).

### 3.2 The GABA in the PDEVs

**Figure 2:**
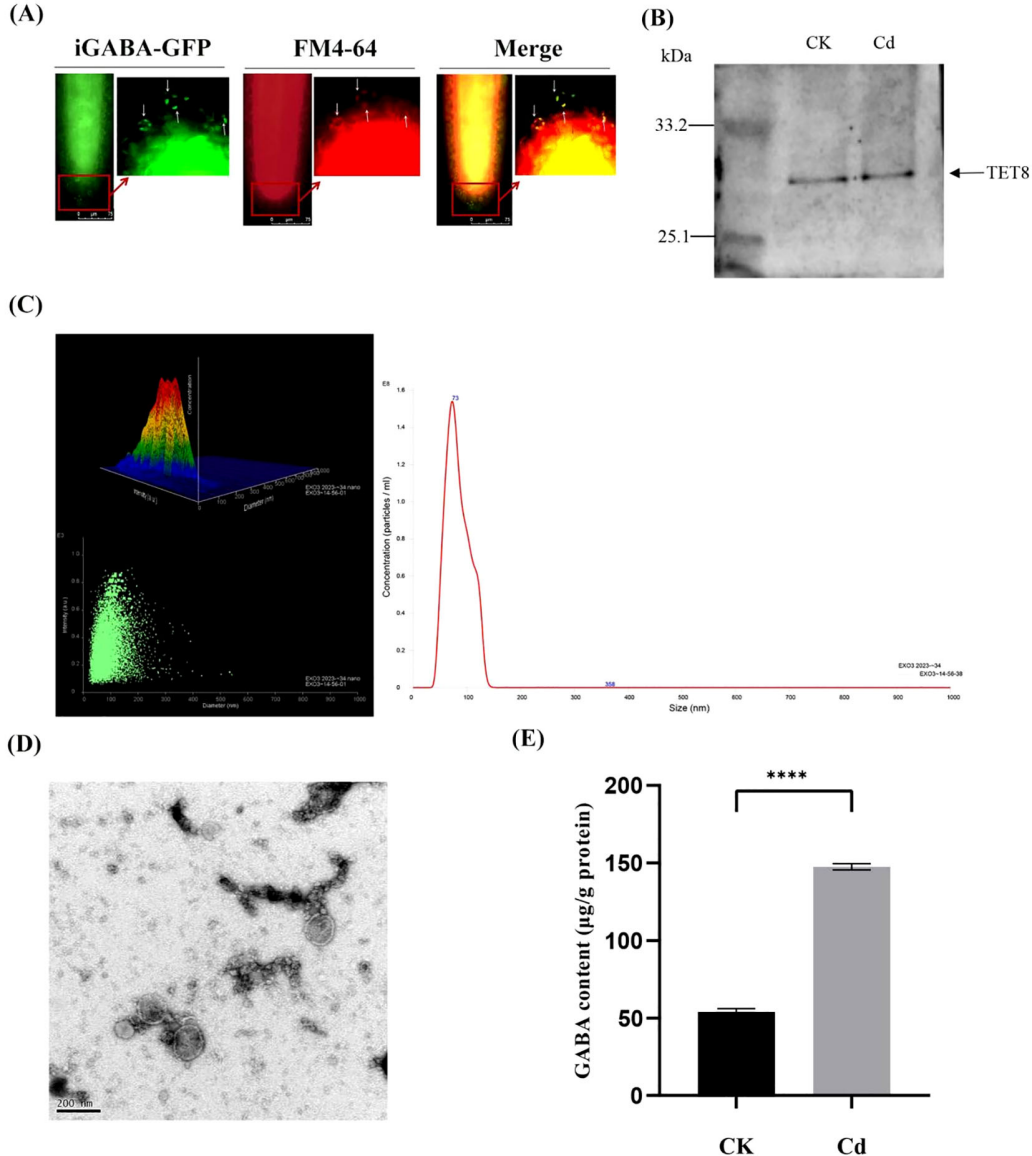
**(A)** iGABA-fluorescence (GFP) and iGABA membrane fluorescence (FM4-64) signal colocalization (Merge) under Cd stress. **(B)** Western blots of proteins in PDEV (CK and Cd) detected using anti-TET8 (tetraspanin 8). **(C)** The size and quantity of PDEVs from iGABA-duckweed were determined using NTA. **(D)** Electron microscopic images of PDEVs from iGABA-duckweed (scale bar, 200 nm). **(E)** GABA content of duckweed PDEVs in control and Cd stress. Significant differences were analyzed using independent samples *t*-test and are indicated by asterisks (****p < 0.001).

To further study GABA’s response during Cd stress, the membrane of iGABA was stained with the FM4-64 dye and then treated with CdCl_2_ for 20 min. Subsequently, the root was observed in two channels (FM4-64; GFP; Merge) using a fluorescence microscope. The results showed obvious vesicular structures in the root cells, and the results of the Merge channel showed that the two fluorescence signals exactly overlapped (**Figure 2A**).

We employed Western blot analysis using a TET8-specific antibody to validate the presence of TET8, a proposed exosome marker, in purified plant exosomes. Western blots of proteins in PDEV (CK and Cd) were detected using anti-TET8 (**Figure 2B**). PDEVs were isolated and analyzed by the NTA assay (**Figure 2C**). The size showed a distribution range of 100 nm average diameter. Additionally, PDEV concentration (particle counts) was determined by NTA as well. The results demonstrated that the majority of isolated exosomes exhibited a diameter distribution predominantly within the range of 0–100 nm, with a distinct peak at 73 nm. This size profile aligns closely with the established criteria for PDEVs (**Figure 2C**). Meanwhile, PDEVs have been found using the transmission electron microscope (**Figure 2D**). The GABA content of duckweed PDEVs under Cd treatment was measured. After 24 h of Cd stress, the GABA content in PDEVs increased to 147.54 μg/g protein, significantly higher than that of the control group—53.98 μg/g protein (**Figure 2E**). A 2.7-fold increase was observed in the GABA content within the PDEVs of duckweed under cadmium stress compared to the control group. This finding is statistically significant (*p* < 0.001), suggesting the role of GABA in response to Cd stress. Furthermore, the expression of the GABA receptor ALMT was studied with or without Cd stress, and the results in **Supplementary Table S2** showed that ALMT was upregulated during Cd stress.

### 3.3 Construction and identification of GAD

**Figure 3:**
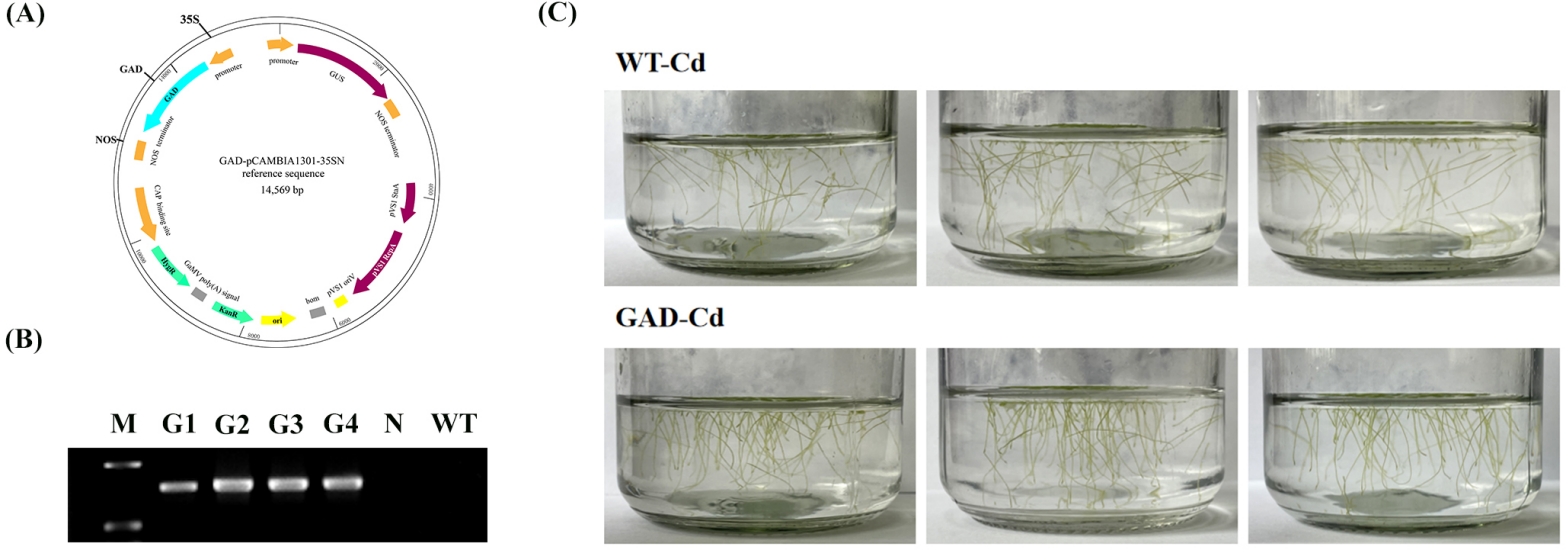
**(A)** Construction of *pCAMBIA-1301****-****GAD*. KanR, kanamycin resistance; HygR, hygromycin resistance; GUS, β-glucuronidase. **(B)** Specific PCR amplification for GAD identification. G1-4, GAD1-4; N, negative control (where water served as the template for PCR); WT, wild type. **(C)** Root abscission in GAD and WT after 24 h of treatment with 50 µM CdCl_2_.

The *GAD*-pCAMBIA1301 vector was constructed and *Agrobacterium tumefaciens* EHA105 was subsequently used to infect duckweed callus (**Figure 3A**). Transgenic duckweed expressing *AtGAD* was successfully obtained using the methods previously described. PCR identification of GAD was performed to test the transgenic duckweed (**Figure 3B**). Notably, *AtGAD* was not amplified in the negative control or WT. These results confirmed the successful transformation of all four transgenic strains. Importantly, GAD showed a significantly lower root abscission rate (15.69%) compared to the WT (29.79%) (**Figure 3C**). The GAD transgenic duckweed shows a significantly lower root abscission rate (15.69%) compared to the wild type (29.79%) under Cd stress. This result was statistically significant and showed that GAD overexpression enhanced cadmium tolerance.

### 3.4 *GAD* overexpression improved chlorophyll content and photosynthesis of duckweed under Cd stress

**Figure 4:**
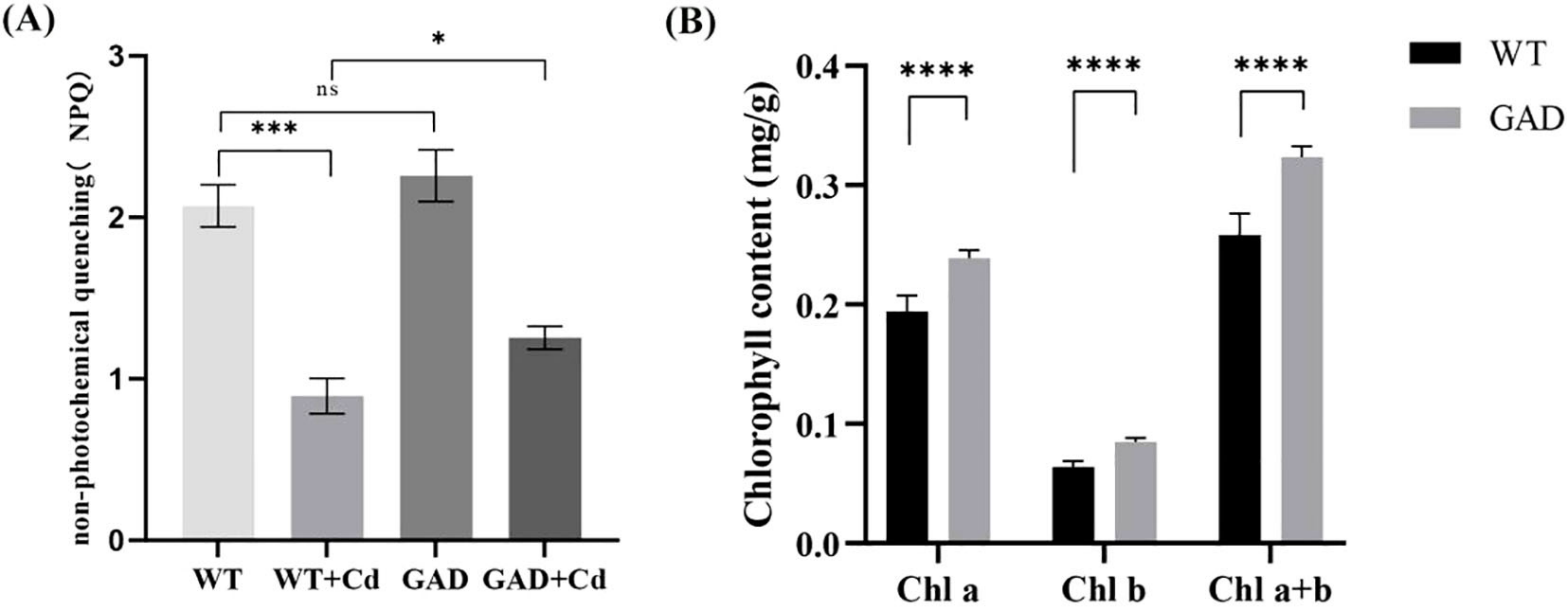
**(A)** The non-photochemical quenching (NPQ) of WT, WT-Cd, GAD, and GAD-Cd. **(B)** The chlorophyll content (Chl a, Chl b, and Chl a+b) of WT and GAD under Cd stress at 48 h “*” indicates a significant difference between treatments using SPSS (*p <0.05,***p < 0.001, ****p < 0.0001).

Non-photochemical quenching (NPQ) in GAD and WT transgenic duckweed under Cd stress was tested, and the results showed that NPQ in GAD duckweed under Cd stress was significantly higher than that in WT duckweed under Cd stress (**Figure 4A**). Chlorophyll plays a vital role in plant photosynthesis. As shown in **Figure 4**, the WT duckweed under Cd stress contained 0.194 mg/g of Chl a, 0.064 mg/g of Chl b, and 0.258 mg/g of Chl a+b. Moreover, GAD contained 0.239 mg/g of Chl a, 0.085 mg/g of Chl b, and 0.324 mg/g of Chl a+b. The results exhibited that compared with the WT, the content of Chl a, Chl b, and Chl a+b in GAD increased by 23.1%, 32.8%, and 25.6%, respectively. These results showed that under Cd stress, the GAD transgenic duckweed exhibited significant increases in chlorophyll a, chlorophyll b, and total chlorophyll content compared to the WT with a statistically significant difference (*p* < 0.0001), showing that GAD overexpression improved the photosynthetic capacity of duckweed under stress. Furthermore, the expression of photosynthesis differential genes and photosynthetic antenna proteins in transcriptome sequencing was analyzed (**Supplementary Table S2**). The results revealed that multiple protein genes associated with photosynthesis were significantly upregulated and that the gene expression of LHCA 3 and LHCB 6 in antenna proteins was 1.534 and 1.692, respectively, enhancing the light energy capture ability of transgenic duckweed.

### 3.5 Determination of protection enzyme activity in GAD

**Figure 5:**
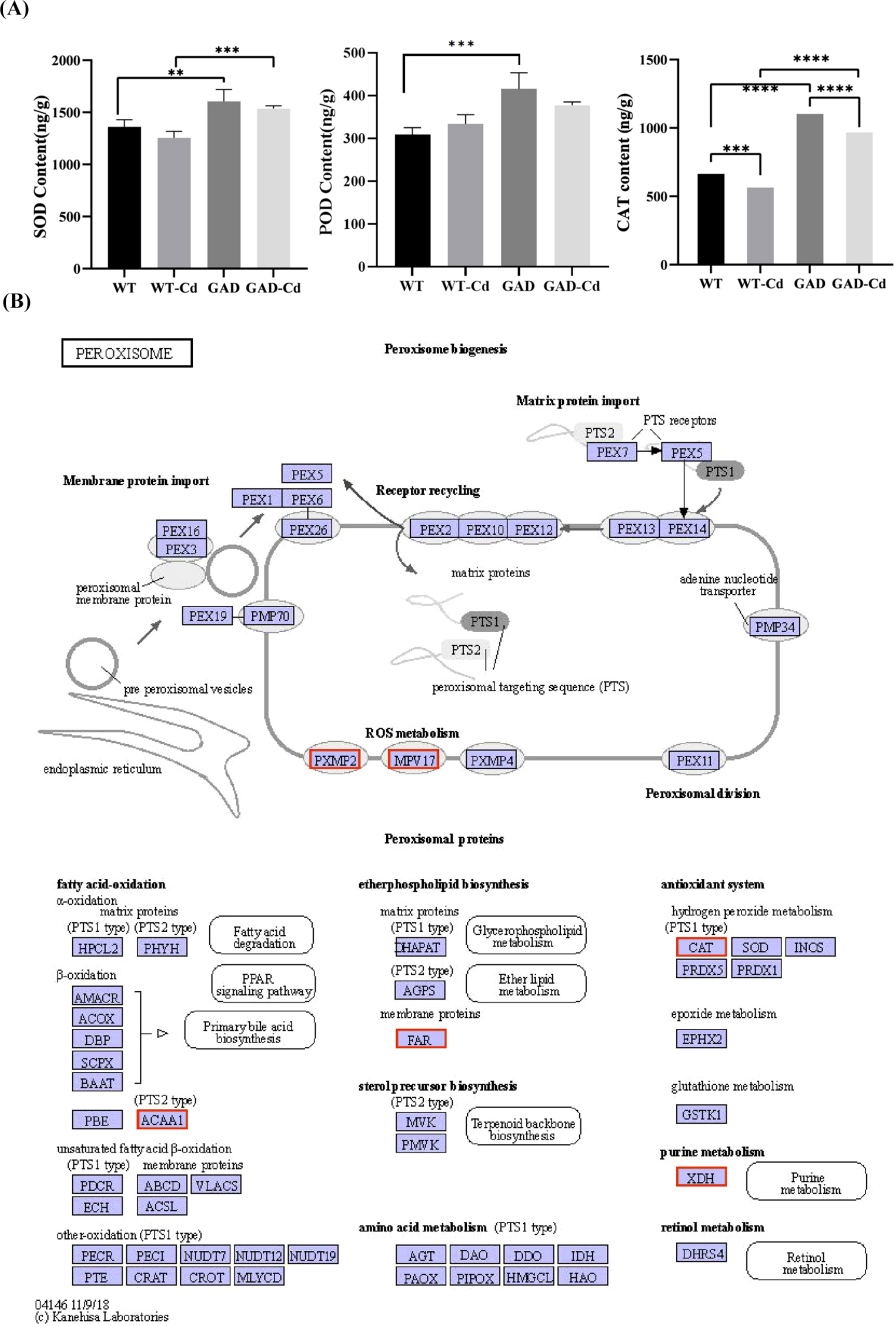
Study of antioxidant capacity and peroxisome gene expression in GAD duckweed during Cd stress. **(A)** Levels of SOD, POD, and CAT in WT and GAD treated with or without 50 μM of Cd for 48 h Significant differences were analyzed using independent samples *t*-test and are indicated by asterisks (** p < 0.01, ***p < 0.001, ****p < 0.0001). **(B)** Changes in peroxisome gene expression under Cd stress. The red box indicates upregulation. Different letters within the same column indicate significant differences between treatments at *p <*0.05 using SPSS.

The antioxidant enzyme activity in WT and GAD duckweed with or without Cd stress was analyzed. The results showed that GAD duckweed exhibited a significant increase in SOD, POD, and CAT levels under Cd stress. Moreover, *GAD* duckweed demonstrated significantly higher levels of SOD and CAT content in comparison to the WT during Cd stress (**Figure 5A**), and these results showed that GAD transgenic duckweed enhanced the antioxidant system, contributing to increased cadmium tolerance. Meanwhile, the expression of the CAT protein gene was significantly upregulated 5.9448 log_2_ fold in the analysis of transcriptome sequencing (**Figure 5B**), which further verified that GAD can enhance the protection of cells from oxidative stress, while demonstrating that overexpressing *AtGAD* in duckweed improved Cd^2+^ tolerance in duckweed again. In **Figure 5B**, gene expression of the mitochondrial inner membrane protein MPV17 and the peroxisomal membrane protein PXMP2 showed significant upregulation in GAD under Cd stress. MPV17 is a mitochondrial protein implicated in reactive oxygen species (ROS) homeostasis and mitochondrial DNA stability. PXMP2 is a key component of the peroxisomal membrane, facilitating the transport of small molecules and ROS-detoxifying enzymes (e.g., CAT) into peroxisomes. These results support the coordinated role of peroxisomal and mitochondrial proteins in Cd tolerance.

### 3.6 Metabolic effects of *GAD* overexpression on GABA

**Figure 6:**
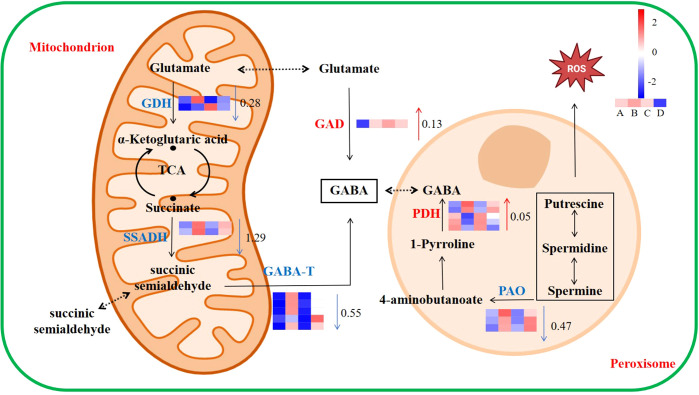
Response of the GABA metabolic pathway to Cd stress in duckweed. The color scale ranges from red (high expression) to blue (low expression), with changes indicated by log_2_ fold change values (**A**: WT, **B**: WT-Cd, **C**: GAD, **D**: GAD-Cd). The color of the arrows indicates upregulation (red) or downregulation (blue) (GAD, glutamate decarboxylase; GABA-T, γ-aminobutyrate-transaminase; SSADH, succinic semialdehyde dehydrogenase; GDH, glutamate dehydrogenase; PDH, pyrroline dehydrogenase; PAO, polyamine oxidase).

The expression of genes related to the GABA metabolism pathway is shown in **Figure 6**. The gene expression of GAD and pyrroline dehydrogenase (PDH) was upregulated compared to the WT with Cd treatment, which increased by 0.13 and 0.05 log_2_ fold change, respectively. Under Cd stress, the gene expression of GABA transaminase (GABA-T), succinic semialdehyde dehydrogenase (SSADH), polyamine oxidase (PAO), and glutamate dehydrogenase (GDH) in GAD was downregulated compared to the WT, which decreased by 0.55, 1.29, 0.47, and 0.28 log_2_ fold change, respectively.

### 3.7 Cd absorption was improved in GAD

**Figure 7:**
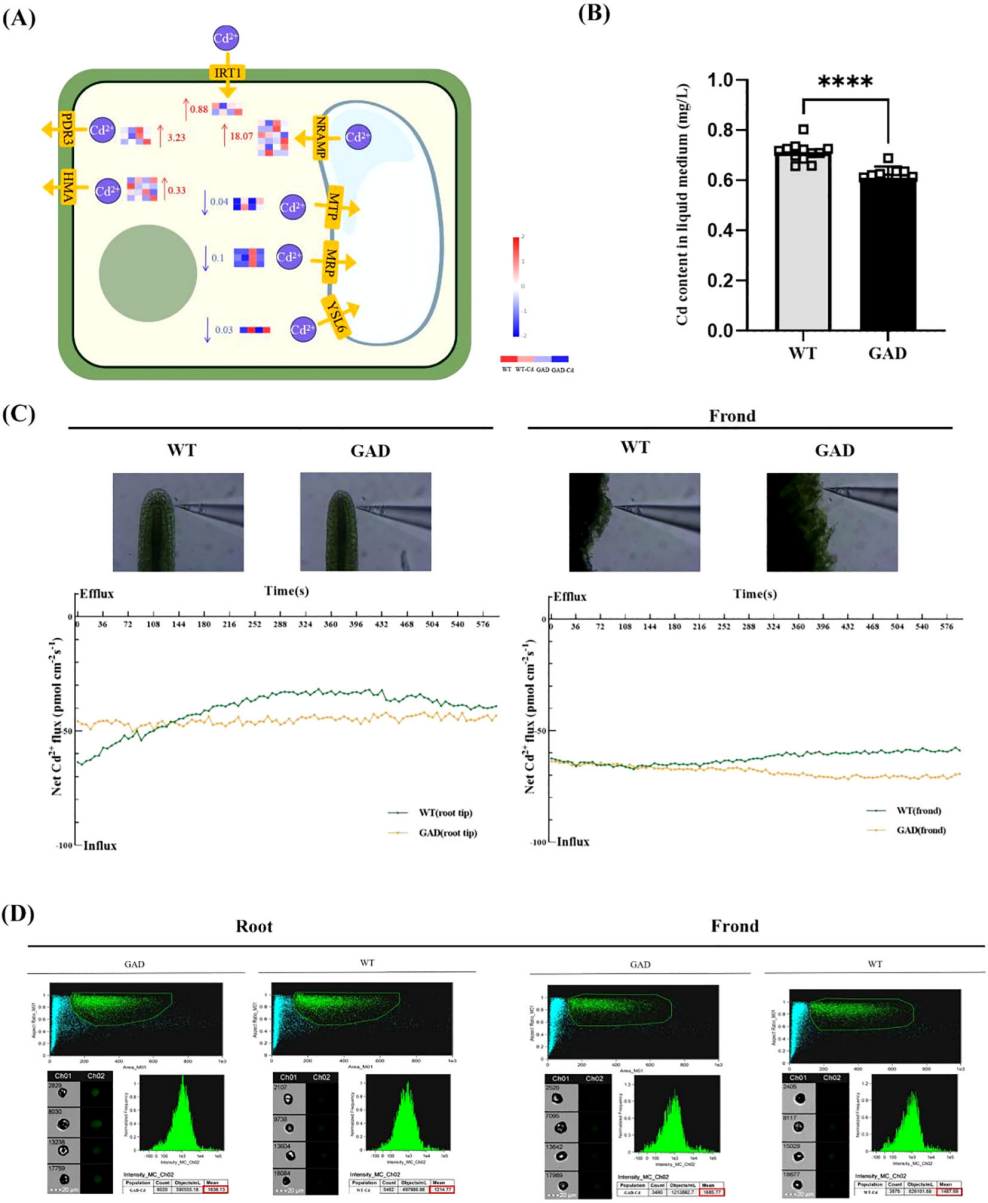
Study of Cd content in water for duckweed culture, Cd^2+^ flux, and Cd uptake in GAD and WT. **(A)** Gene expression of the Cd^2+^ transporter protein in WT and GAD with or without CdCl_2_. The color scale ranges from red (high expression) to blue (low expression), with changes indicated by log_2_ fold change values. The color of the arrows indicates upregulation (red) or downregulation (blue). (HMA, heavy metal ATPase natural; RAMP, resistance-associated macrophage protein; MTP, metal tolerance protein; MRP, multidrug resistance-associated protein; YSL6, metal-nicotianamine transporter YSL6; ZIP, zinc transporter; IRT1, Fe^2+^ transport protein 1; PDR3, pleiotropic drug resistance protein). **(B)** Cd content in the water cultured with duckweed (WT/GAD). Significant differences were analyzed using independent samples *t*-test and are indicated by asterisks (****p < 0.001). **(C)** Net Cd^2+^ flux in the roots and leaves of WT and GAD, measured using NMT after treatment with 50 µM of CdCl_2_ for 30 min (negative values represent influx; absolute values represent flow velocity). **(D)** Cd fluorescence intensity in protoplasts from roots and fronds analyzed using the FlowSight system at 488 nm. Both GAD and WT were soaked in 50 µM of Cd for 24 h Leadmium™ Green AM was used to stain their protoplasts. Ch 01 is bright field, and Ch 02 is 488 nm of excitation light, with a scale bar of 20 µm. The protoplasts, framed in green, were selected to analyze fluorescence intensity.

The expression of Cd transport relative genes was studied. The results in **Figure 7A** showed that the expression of PDR3, HMA, IRT1, and NRAMP was increased by 0.33, 3.23, 0.88, and 18.07 log_2_ fold times during Cd stress in GAD duckweed compared to WT duckweed. The Cd content in the liquid medium cultured with WT or GAD under 72 h of Cd stress was estimated by ICP. As shown in **Figure 7B**, the Cd content in the liquid medium of WT duckweed was 0.71 mg/L, while the Cd content in the liquid medium cultured with GAD was 0.61 mg/L. Obviously, GAD demonstrates a stronger capability of removing cadmium from water. This result showed a significant decrease in cadmium content in the liquid medium of GAD transgenic duckweed compared to the wild type, indicating a greater cadmium removal ability in transgenic plants. Meanwhile, real-time Cd^2+^ fluxes in the roots and fronds of WT and GAD were measured in a Cd^2+^ solution using the NMT assay after 50 µM of CdCl_2_ treatment (**Figure 7C**). In the roots, the Cd^2+^ influx velocity of GAD was lower compared to the WT before 132 s. The Cd^2+^ influx velocity of GAD was higher compared to the WT after 132 s. In the fronds, the influx velocity of Cd^2+^ in the duckweed fronds was roughly the same in the two groups before 132 s. After 132 s, the Cd^2+^ influx velocity of GAD was higher than the WT. Furthermore, Cd fluorescence intensity in the roots and fronds of WT and GAD was detected after Cd staining using flow cytometry (**Figure 7D**). The results revealed that Cd^2+^ fluorescence intensity in the roots of GAD (mean = 1,636.13) was higher than that of the WT (mean = 1,214.77), and Cd^2+^ fluorescence intensity in the fronds of GAD (mean = 1,685.77) was higher than that of the WT (mean = 1,487.69). These results showed that Cd accumulation ability was enhanced in GAD.

## 4 Discussion

### 4.1 GABA signaling via extracellular vesicles in plant communication

Previous investigations have illuminated the capacity of GABA to accumulate in response to diverse abiotic stresses, including salinity, drought, and heat, where it functions as a signaling molecule or osmoticum to mitigate stress-induced damage (Xu et al., 2021). This upregulation suggests that GABA functions as a signaling molecule in response to stress, potentially aiding in stress tolerance and adaptation mechanisms. In animals, GABA is efficiently packaged and released from the ventral tegmental area (VTA) through independent pools of vesicles, facilitating interneuronal communication (Root et al., 2014; Mondoloni and Mameli, 2022). However, the mechanisms underlying GABA’s intercellular transfer and signaling in plants remain elusive. In this study, we report the discovery of GABA’s role in stress response through its release in the form of extracellular vesicles in duckweed (**Figure 2**). Our results showed that under Cd stress, duckweed packaged and released GABA via extracellular vesicles, which served as a signaling molecule. This vesicle-mediated GABA transport parallels the acetylcholine (Ach) signaling mechanism we previously identified in duckweed extracellular vesicles (Yang L. et al., 2023), suggesting a conserved role for vesicular trafficking in plant stress communication. After treatment with Cd for 24 h, the GABA content in extracellular vesicles was 2.7 times higher than that of CK duckweed. The selectivity of GABA packaging into vesicles may involve specific lipid–protein interactions or cargo-sorting machinery, as observed in animal exosome biogenesis (Marvin et al., 2019). Future studies should explore whether GABA-loaded vesicles are selectively internalized by neighboring cells or released into the rhizosphere to modulate microbial communities under Cd stress (Ramesh et al., 2015).

### 4.2 GAD overexpression: benefits and limitations

GAD is critical for plant stress responses. Xu et al. found that cytosolic GABA signals generated by the enzyme GAD2 play a pivotal role in improving water use efficiency and drought tolerance (Xu et al., 2021). In this study, overexpressing GAD in duckweed significantly enhanced Cd tolerance (**Figure 3**), accompanied by modified expression of GABA metabolism-related enzymes. However, transcriptomic analysis revealed potential off-target effects, including altered expression of genes involved in glutamate metabolism (e.g., GLUTAMATE SYNTHASE 1, log_2_ FC = 3.2) and redox homeostasis (**Supplementary Table S3**). These findings suggest that GAD overexpression may perturb broader metabolic networks, necessitating targeted metabolomic profiling to assess trade-offs. To mitigate biosafety risks, GAD transgenic duckweed should be confined to controlled environments such as wastewater treatment tanks, as gene flow via vegetative propagation could lead to unintended genetic introgression in natural ecosystems. This finding provides evidence for GABA’s response and the functional role of GAD under Cd stress.

### 4.3 Balancing GABA and glutamate signaling

Under these circumstances, GABA and Glu emerge as crucial metabolites. Produced in the cytosol, GABA and Glu serve as a bypass for several reactions of the TCA cycle, particularly during times of metabolic disruption. The more, the better? Probably not. Studies showed that rapid, long-distance Glu signaling responds in *Arabidopsis* during damage (Toyota et al., 2018). The concentration of glutamate plays a pivotal role in its signaling capacity. Within a certain range, elevated Glu levels can stimulate neurons. However, excessive Glu levels can be detrimental, leading to neuronal excitotoxicity in animals (Li et al., 2020). Moreover, our study showed similar results in plants (Yang et al., 2020). Regarding GABA, studies showed that exogenous application improved stress tolerance, while in our previous studies, we found that a higher concentration of GABA enhanced Cd damage in duckweed. This finding suggests the delicate balance that must be maintained in GABA signaling to ensure the signal response and the balance of GABA metabolism for plants. The discovery of the GABA signaling pathway in plants provides new avenues for exploring plant adaptation and stress tolerance mechanisms. Meanwhile, overexpression of GAD in duckweed demonstrates the potential of genetic engineering to enhance plant resistance to environmental stressors, with important implications for phytoremediation and ecological restoration efforts. In duckweed, we propose that vesicle-mediated GABA release fine-tunes intercellular stress signals without overwhelming cellular redox balance. This novel observation expands our understanding of GABA’s functions beyond the animal kingdom and highlights its role in mediating the communication between duckweed and its environment through vesicular trafficking.

### 4.4 Implications for phytoremediation

The discovery of vesicle-based GABA signaling and GAD-enhanced Cd tolerance opens new avenues for phytoremediation. Transgenic duckweed could be deployed in Cd-contaminated wastewater systems, leveraging its rapid biomass accumulation and metal uptake efficiency. However, field applications require rigorous risk assessments, including monitoring for unintended ecological impacts. Future work should optimize GABA flux engineering while minimizing metabolic perturbations, potentially through tissue-specific promoter expression systems. The discovery of GABA release via extracellular vesicles (EVs) in duckweed under Cd stress suggests novel ecological implications for plant–microbe interactions and community dynamics. Based on our findings, GABA-loaded EVs may act as signaling molecules that modulate rhizosphere microbial communities under stress conditions.

## Data Availability

The datasets presented in this study can be found in online repositories. The names of the repository/repositories and accession number(s) can be found in the article/**Supplementary Material**.
