# Supplementary figures and images for: Extracellular vesicle GABA responds to cadmium stress, and GAD overexpression alleviates cadmium damage in duckweed
Source: Front Plant Sci. 2025 Mar 18;16:1536786. doi: 10.3389/fpls.2025.1536786 (PMC11959025; doi:10.3389/fpls.2025.1536786)

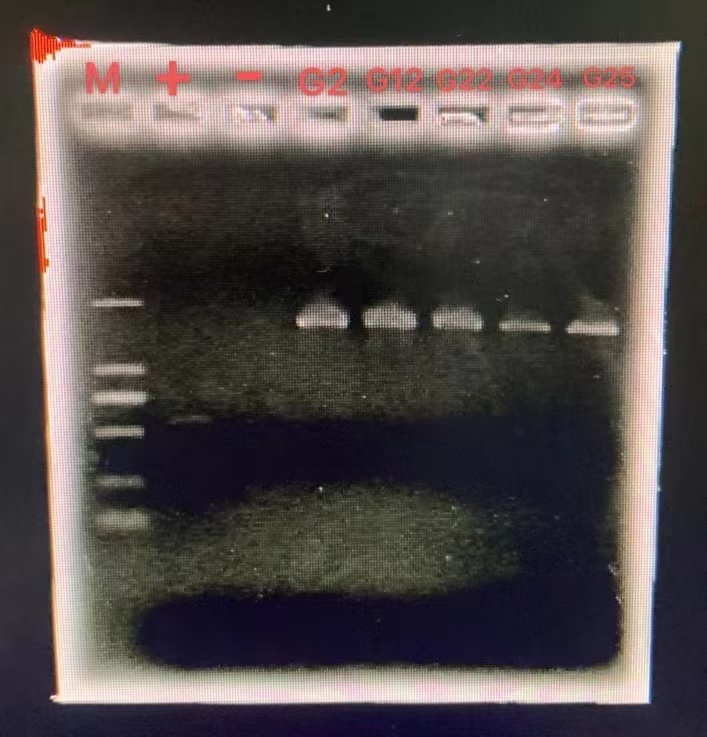

Supplement: Supplementary file 1 [file Image1.jpeg]

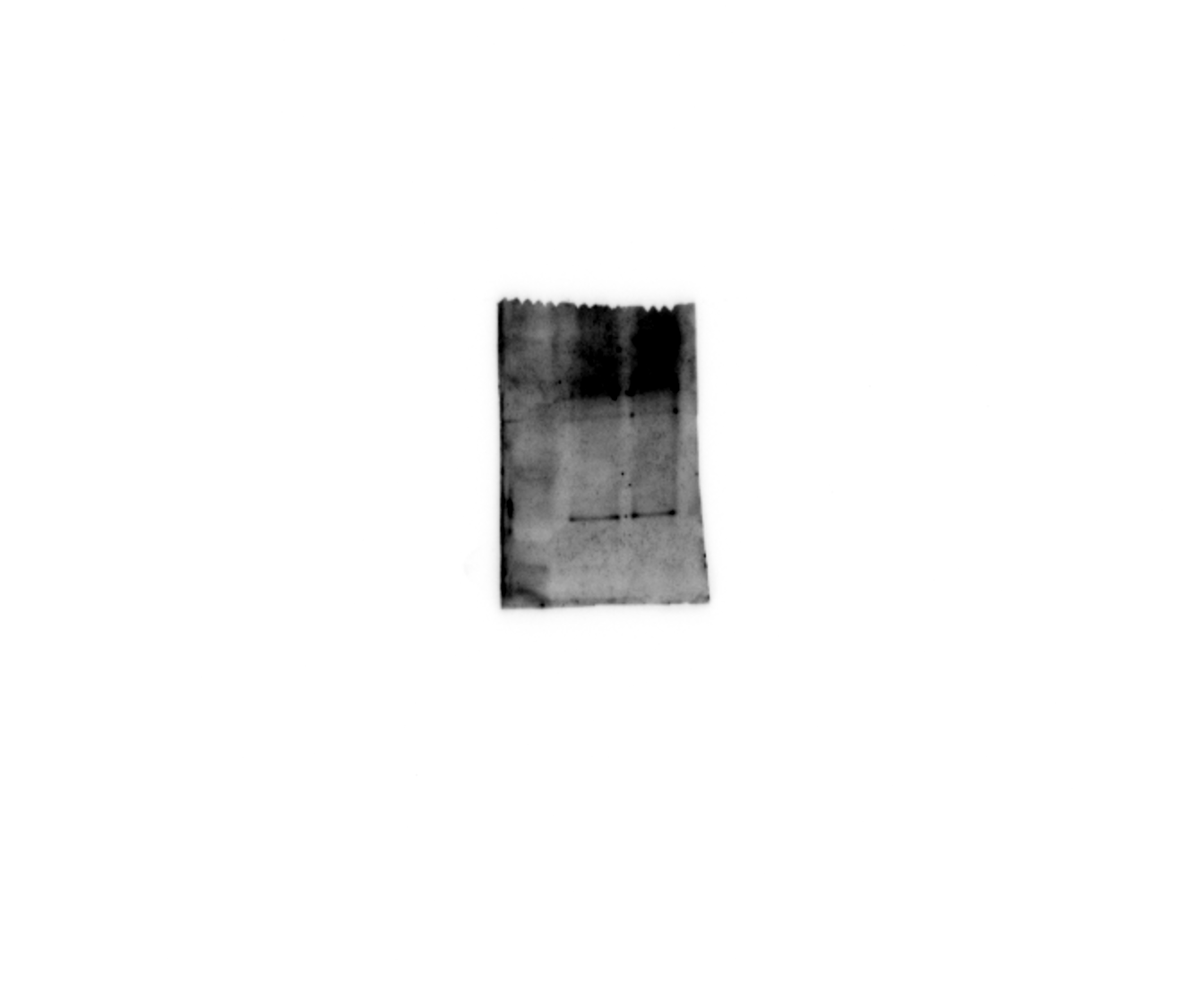

Supplement: Supplementary file 2 [file Image2.tif]

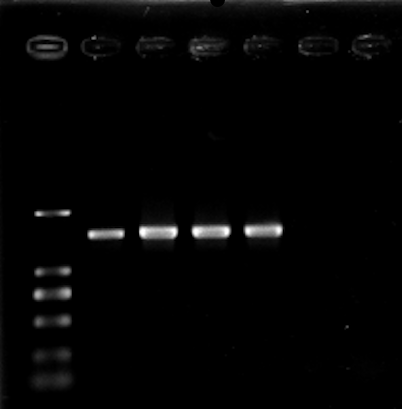

Supplement: Supplementary file 3 [file Image3.tif]
