# Supplementary material for: Extracellular vesicle GABA responds to cadmium stress, and GAD overexpression alleviates cadmium damage in duckweed
Source: Front Plant Sci. 2025 Mar 18;16:1536786. doi: 10.3389/fpls.2025.1536786 (PMC11959025; doi:10.3389/fpls.2025.1536786)
Supplement: Supplementary file 4 [file Table1.docx]

| Table S1 The expression of GAD in transgenic and WT duckweed | | | | | | | |
| --- | --- | --- | --- | --- | --- | --- | --- |
| Gene_id | GAD_readcount | WT_readcount | log2FoldChange | pvalue | padj | Gene Length | NT Description |
| Cluster-10487.21229 | 292.65 | 5.79 | 5.67 | 1.5048e-08 | 5.1815e-06 | 2396 bp | Arabidopsis thaliana glutamate decarboxylase (GAD), mRNA |

| Table S2 The expression of ALMT in transgenic and WT duckweed | | | | | | |
| --- | --- | --- | --- | --- | --- | --- |
| gene_id | Annotation | Cd_readcount | CK_readcount | log2FoldChange | pval | padj |
| Cluster-7365.100029 | Aluminium activated malate transporter | 18.57 | 1.48 | 3.5948 | 0.00086335 | 0.0055902 |
| Cluster-7365.16679 | aluminum-activated malate transporter 5-like isoform X1 [Ananas comosus] | 230.48 | 80.88 | 1.5154 | 0.00023419 | 0.0017028 |
| Cluster-7365.16678 | Aluminum-activated malate transporter 9 | 239.81 | 14.12 | 4.0928 | 0.00059464 | 0.0039898 |

| Table S3 Changes in gene expression levels related to photosynthesis and antenna proteins | | | | | | | | | | | |
| --- | --- | --- | --- | --- | --- | --- | --- | --- | --- | --- | --- |
| Description | gene id | GAD readcount | WT readcount | log_2_FoldChange | P-value | padj | GAD-Cd readcount | WT-Cd readcount | log_2_FoldChange | P-value | padj |
| photosystem II oxygen-evolving enhancer protein 3 (psbQ) | Cluster-10487.10585 | 1316.88 | 2038.14 | -0.630 | 0.00030909 | 0.016007 | 784.90 | 373.17 | 1.072 | 0.032 | 0.924 |
| photosystem II 22kDa protein (psbS) | Cluster-10487.12967 | 34336.75 | 50701.92 | -0.562 | 0.00057178 | 0.026103 | 20909.56 | 8117.55 | 1.365 | 0.007 | 0.632 |
| photosystem II Psb27 protein (psb 27) | Cluster-10487.12473 | 6020.80 | 8706.76 | -0.532 | 0.0018328 | 0.060809 | 1612.08 | 650.00 | 1.310 | 0.041 | 0.961 |
| photosystem I subunit III (psaF) | Cluster-10487.13871 | 28764.98 | 43125.01 | -0.584 | 0.00012263 | 0.0078142 | 3655.19 | 1416.51 | 1.368 | 0.006 | 0.603 |
| photosystem I subunit V (psaG) | Cluster-10487.12888 | 14610.67 | 20377.23 | -0.480 | 0.0016285 | 0.055484 | 2252.18 | 722.66 | 1.640 | 0.012 | 0.812 |
| photosystem I subunit X (psaK) | Cluster-10487.13026 | 42174.15 | 62017.06 | -0.556 | 0.00016485 | 0.0098808 | 7916.97 | 3090.61 | 1.357 | 0.030 | 0.917 |
| photosystem I subunit PsaO (psaO) | Cluster-10487.13357 | 36970.69 | 54057.08 | -0.548 | 0.0028735 | 0.083413 | 6826.57 | 2312.00 | 1.562 | 0.020 | 0.905 |
| light-harvesting complex I chlorophyll a/b binding protein 1 (LHCA 1) | Cluster-10487.13206 | 17915.67 | 26701.82 | -0.576 | 0.00027982 | 0.014727 | 1174.55 | 358.58 | 1.711 | 0.020 | 0.905 |
| light-harvesting complex I chlorophyll a/b binding protein 3 (LHCA 3) | Cluster-10487.13413 | 26288.73 | 40629.46 | -0.628 | 2.76E-05 | 0.0024182 | 4314.90 | 1489.93 | 1.534 | 0.033 | 0.928 |
| light-harvesting complex I chlorophyll a/b binding protein 4 (LHCA 4) | Cluster-10487.13388 | 55110.51 | 86168.20 | -0.645 | 0.00014116 | 0.008753 | 4127.21 | 1755.30 | 1.233 | 0.014 | 0.848 |
| light-harvesting complex II chlorophyll a/b binding protein 3 (LHCB 3) | Cluster-10487.13166 | 36151.47 | 66116.78 | -0.871 | 3.15E-07 | 6.88E-05 | 1410.03 | 516.77 | 1.448 | 0.007 | 0.626 |
| light-harvesting complex II chlorophyll a/b binding protein 4 (LHCB 4) | Cluster-10487.13012 | 70947.47 | 128982.92 | -0.862 | 7.31E-07 | 0.00013598 | 10746.94 | 5934.60 | 0.857 | 0.032 | 0.928 |
| light-harvesting complex II chlorophyll a/b binding protein 5 (LHCB 5) | Cluster-10487.12014 | 1654.10 | 2793.93 | -0.756 | 0.0020823 | 0.065718 | 346.60 | 199.99 | 0.793 | 0.793 | 0.026 |
| light-harvesting complex II chlorophyll a/b binding protein 6 (LHCB 6) | Cluster-10487.13058 | 20671.15 | 35198.08 | -0.768 | 4.47E-06 | 0.00058756 | 1630.26 | 504.47 | 1.692 | 0.002 | 0.270 |
